# Asthma, Airflow Obstruction, and Eosinophilic Airway Inflammation Prevalence in Western Kenya: A Population-Based Cross-Sectional Study

**DOI:** 10.3389/ijph.2023.1606030

**Published:** 2023-08-17

**Authors:** Neelima Navuluri, David Lagat, Joseph R. Egger, Elcy Birgen, Lameck Diero, David M. Murdoch, Nathan Thielman, Peter S. Kussin, Loretta G. Que, Devon Paul

**Affiliations:** ^1^ Division of Pulmonary, Allergy and Critical Care, Duke University School of Medicine, Durham, NC, United States; ^2^ Duke Global Health Institute, Duke University, Durham, NC, United States; ^3^ Department of Medicine, Moi University School of Medicine, Eldoret, Kenya; ^4^ Division of Infectious Disease, Duke University School of Medicine, Durham, NC, United States; ^5^ Division of Pulmonary and Critical Care, Mt. Sinai Hospital Medical Center, Chicago, IL, United States; ^6^ Department of Medicine, Rosalind Franklin School of Medicine, North Chicago, IL, United States

**Keywords:** asthma, chronic obstructive pulmonary disease, Kenya, spirometry, cross-sectional study, epidemiology, pulmonary disease, airway inflammation

## Abstract

**Objectives:** Determine the prevalence of airway disease (e.g., asthma, airflow obstruction, and eosinophilic airway inflammation) in Kenya, as well as related correlates of airway disease and health-related quality of life.

**Methods:** A three-stage, cluster-randomized cross-sectional study in Uasin Gishu County, Kenya was conducted. Individuals 12 years and older completed questionnaires (including St. George’s Respiratory Questionnaire for COPD, SGRQ-C), spirometry, and fractional exhaled nitric oxide (FeNO) testing. Prevalence ratios with 95% confidence intervals (CIs) were calculated. Multivariable models were used to assess correlates of airflow obstruction and high FeNO.

**Results:** Three hundred ninety-two participants completed questionnaires, 369 completed FeNO testing, and 305 completed spirometry. Mean age was 37.5 years; 64% were women. The prevalence of asthma, airflow obstruction on spirometry, and eosinophilic airway inflammation was 21.7%, 12.3% and 15.7% respectively in the population. Women had significantly higher SGRQ-C scores compared to men (15.0 vs. 7.7). Wheezing or whistling in the last year and SGRQ-C scores were strongly associated with FeNO levels >50 ppb after adjusting for age, gender, BMI, and tobacco use.

**Conclusion:** Airway disease is a significant health problem in Kenya affecting a young population who lack a significant tobacco use history.

## Introduction

Lower respiratory disease and chronic obstructive pulmonary disease (COPD) have risen to become leading causes of morbidity and mortality worldwide [1]. About 77% of these deaths are thought to occur in low and middle-income countries (LMICs) [2]. The Global Burden of Disease Study estimated that in 2019 that there were over 26 million people affected by asthma and the Global Action Network found that prevalence of asthma symptoms ranged from 6.6% to 11% with wide variation by age and region of the world [1, 3]. However, population-based estimates of the prevalence of chronic lung diseases such as asthma and COPD in East Africa are limited [4, 5].

A systematic review of COPD prevalence in sub-Saharan Africa identified nine studies, of which only one, FRESH AIR, has been conducted in East Africa [6]. It found a COPD prevalence of 16.2% among participants age 30 or older in Uganda [7]. The International Study of Asthma and Allergies in Childhood (ISAAC) [8] and the Global Initiative for Asthma (GINA) [9] have made large strides in our understanding of the rising prevalence of asthma worldwide. Yet, only the ISAAC studies assessed asthma prevalence in Kenya using questionnaires, finding rates in school-aged children of 12.6% in rural areas and 15.4% in urban areas [8, 10]. Similar studies among adolescents and adults in Kenya and studies utilizing spirometry or eosinophilic airway inflammation to diagnose airway disease have not been published.

Furthermore, drivers for the development of airway disorders in LMICs are poorly understood. Tobacco smoke and occupational exposure are thought to be potentially less common in LMICs. Biomass fuels, which are used for cooking in as many as 95% of households across 18 African countries, have been proposed as an important risk factor, especially among women [11]. Additional risk factors include high rates of HIV, tuberculosis, childhood respiratory infections, and socioeconomic factors such as malnutrition and poverty [12–16].

Thus, we sought to primarily quantify the prevalence of asthma, airflow obstruction on spirometry, and eosinophilic airway inflammation (henceforth, collectively referred to as airway disease) in the Eldoret area of Uasin Gishu County, Kenya. We also sought to assess demographic, clinical, and environmental factors associated with the prevalence of airway disease and health-related quality of life.

## Methods

### Study Design

We conducted a cluster-designed, cross-sectional study which took place between August 2016 and August 2017. Uasin Gishu is home to Eldoret, the 4th largest metropolitan area in Kenya. With a population of 1,163,186 in 2019 across urban, peri-urban, and rural areas, a poverty incidence of 33.9%, and average highest education level of primary school among adults, Uasin Gishu afforded a unique opportunity to investigate airway disorders across geographical and socioeconomic gradients [17, 18]. It is comprised of 47 administrative locations which formed the basis of our sampling procedures.

Participants were identified for eligibility screening using a three-stage modified expanded program on immunization (EPI) cluster-randomized, sampling protocol which helps minimize sampling bias [19]. This protocol has been employed in several population-based prevalence studies [20–22]. Individuals age 12 and above were eligible for participation if they resided in Uasin Gishu at the time of enrollment. Exclusion criteria included contraindications to spirometry or bronchodilator administration per American Thoracic Society (ATS) and European Respiratory Society (ERS) guidelines (e.g., acute myocardial infarction within the last 1 week; known abdominal or thoracic aortic aneurysm; brain, thoracic or abdominal surgery within the last 4 weeks; eye surgery within the last week; massive hemoptysis within the last 4 weeks) [23].

A subset of 19 administrative subunits (i.e., primary sampling unit, PSU) encompassing and surrounding Eldoret were selected for recruitment based on proximity to the city center (Supplementary Appendix SA2). Using ArcGIS software (version 10.5, ESRI, Redlands, CA), a random GPS point was dropped within each of the 19 PSUs and served as the initial point of contact. The study team used portable GPS units to get as close to this GPS point as physically and safely possible. Once there, a 360-degree assessment was conducted to find the closest dwelling (i.e., secondary sampling unit, SSU) to the starting point. This served as the first dwelling approached for enrollment. The next dwelling was identified by flipping a coin to determine whether to proceed left or right from the first dwelling, and rolling a die to determine how many dwellings to pass prior to attempting enrollment again. This process was repeated until 7 dwellings per GPS point were enrolled. To ensure the protocol was strictly adhered to, the principal investigator (DP) regularly accompanied the study team.

### Procedures

All members of each dwelling meeting eligibility criteria were approached for participation in the study. The study, including risks and benefits, were discussed in detail with participants in English, Kiswahili, or Kalenjin, according to individual preference. Participants signed an informed consent form in their preferred language, or in case of illiteracy, thumb-printed, and the form was signed by a witness. All study procedures were approved by the ethical review boards at Duke University (Pro00070184), Moi University (IREC/2016/07), and the Kenyan National Commission for Science, Technology, and Innovation (NACOSTI/P/16/25171/13266).

Participants completed a demographics questionnaire, clinical assessment, and St George’s Respiratory Questionnaire for COPD (SGRQ-C) [20–22]. A specific question about wheezing or whistling in the last 12 months was included as part of the clinical assessment as it has been validated as a measure of asthma in children and adolescents through ISAAC and in adults through European Community Respiratory Health Survey (ECRHS) and International Union Against Tuberculosis (IUAT) studies [8, 24–28]. Additional questions regarding respiratory symptoms from ISAAC were also included. The SGRQ-C, a modified version of the St. George’s Respiratory Questionnaire (SGRQ), is a validated survey focused on symptoms most commonly identified by patients with obstructive lung diseases. It provides high quality data in a shorter time than the original version. Scores were adjusted to make them directly comparable to the SGRQ and compared using a clinically significant difference of 4 units [29, 30]. All questionnaires were translated from English to Kiswahili by our research team and back-translated to English to verify accuracy by a research coordinator. Translated questionnaires were pilot tested with volunteer research staff and nurses to ensure translations were accurate and easy to understand.

Spirometry was conducted using a CareFusion MicroLoop spirometer, calibrated according to manufacturer recommendations, in accordance with ATS/ERS guidelines [23]. Participants with an FEV1/FVC ratio <70 as determined by the spirometry device interpretation completed post-bronchodilator spirometry after administration of 400 micrograms of salbutamol via metered dose inhaler. Fraction of exhaled nitric oxide (FeNO) measurements were performed using an Aerocrine Niox Vero machine in accordance with manufacturer recommendations. Single measurements were obtained and stored on the Niox Vero device and recorded electronically.

Data were captured using Qualtrics survey software (Version 2017, Qualtrics, Provo, UT) on a tablet computer. Data were entered a single time and 20% of entries were checked for internal consistency and accuracy, including verifying measurements from equipment records. Participants with abnormal spirometry per device interpretation or significant respiratory symptom burden on questionnaires were referred to subspecialty care. Participants were compensated with 100KES (∼$1USD) mobile phone credit vouchers or cookies per preference.

### Definitions

The presence of whistling or wheezing in the previous 12 months was used as a proxy definition for asthma. This question has been used as a proxy definition for asthma in several studies across various settings and has been validated in children through ISAAC studies and in adults through ECRHS and IUAT studies [25–28, 31–33]. Airflow obstruction was defined as an forced expiratory volume in one second (FEV_1_) to forced vital capacity (FVC) ratio below lower limit of normal (LLN; 5^th^ percentile of predicted) as calculated by the Global Lung Initiative (GLI) 2012 Data Conversion software (Version 1.3.4) [34, 35]. Data on the most accurate prediction equations in a Kenyan population are lacking, so GLI-Other equations were used to re-calculate predicted values based on data from South Africa [36]. Global Initiative for Chronic Obstructive Lung Disease (GOLD) criteria were used to classify severity. An FVC < LLN in participants with a normal FEV_1_:FVC ratio was interpreted as possibly representing lung restriction. Eosinophilic airway inflammation was defined as an FeNO measurement of >50 parts per billion (ppb).

### Statistical Analysis

The study was originally powered on a sample size of 544 participants to estimate a sample prevalence of obstructive airflow obstruction of 17% (95% CI 13.4–21.4) and a design effect of 1.5. Given a realized sample of 305 participants, and assuming the same population prevalence of 17% and a design effect of 1.5, we estimate being able to recover 17% prevalence with a 95% precision of 12.3%–23.2%. Prevalence was defined as the proportion of those individuals who had an FEV_1_:FVC ratio <LLN. Standard errors were adjusted for the complex sampling design using the “svy” command in Stata (version 17, StataCorp, College Station, TX) [37, 38]. Specifically, a Taylor series linearization method was used, assigning each study participant to one of 19 administrative subunits (PSU, sampled without replacement) and household dwelling. Prevalence ratios and 95% confidence intervals for asthma, airway obstruction on spirometry, and eosinophilic airway inflammation were calculated using a log-binary generalized estimating equation (GEE) regression model, accounting for clustering at the household level. Of note, there was very little residual clustering at the village level after accounting for household level, so only the latter was included in regression analyses. Univariable models were fit separately for each independent variable. A multivariable model was fit and included independent variables that were thought, *a priori*, to be common causes of both the other independent variables and the outcome. Missing data were assumed to occur completely at random and were excluded from the final analyses. All analyses were performed using Stata software. Given the exploratory nature of the study, no statistical significance testing was performed.

## Results

Of the 399 individuals screened, 392 participants agreed to participate and underwent questionnaires and spirometry. Of the 392 participants, 305 (77%) were able to provide acceptable spirometry by ATS/ERS criteria (Supplementary Appendix SA1) and 369 (94.1%) were able to provide FeNO results. There was little difference between those with acceptable spirometry and the 87 participants without acceptable spirometry in terms of age, sex, and tobacco use.

Of the 392 participants who completed demographics and questionnaires, mean age was 37.48 (SD 19.48); 64% were women (Table 1). Population-level data for Uasin Gishu county from the Kenyan National Census in 2019 as compared to our sample population are shown in Supplementary Appendix SA3 [17]. Compared to Uasin Gishu county, our sample is more female (64% vs. 50%) with a higher proportion of older participants. Given the imbalance in sex, we stratified our results by sex. Tobacco use was more common among men. A higher proportion of women reported using biomass fuels for over half of their household needs, with firewood being the primary biomass fuel source. On self-report, 7 (2.2%) participants had HIV and 5 (1.3%) had prior tuberculosis. Mean vital signs were within normal physiologic ranges (Supplementary Appendix SA4), though 15 (3.8%) participants had a pulse oximetry reading of ≤90% on room air, of which 12 (80%) were women.

**TABLE 1:** Demographic characteristics of study population (Eldoret, Kenya, 2017).

	Male	Female	Total
	(N = 140)	(N = 252)	(N = 392)
Age	35.13 (1.49)	38.79 (1.22)	37.48 (1.03)
Range	12–91	12–94	12–94
BMI	19.83 (0.43)	22.52 (0.41)	21.56 (0.39)
Wage per day in US Dollars	3.05 (0.53)	1.68 (0.28)	2.21 (0.28)
Last level of schooling completed			
None	6 (4.3%)	40 (15.9%)	46 (11.7%)
Primary	77 (55.0%)	124 (49.2%)	201 (51.3%)
Secondary	37 (26.4%)	67 (26.6%)	104 (26.5%)
Diploma	17 (12.1%)	16 (6.3%)	33 (8.4%)
Degree	3 (2.1%)	5 (2.0%)	8 (2.0%)
Tobacco Smoking Status			
Never Smoker	109 (77.9%)	227 (90.1%)	336 (85.7%)
Former Smoker	15 (10.7%)	22 (8.7%)	37 (9.4%)
Current Smoker	16 (11.4%)	3 (1.2%)	19 (4.8%)
# of Tobacco Sticks per Day*	3.8 (3.9)	2.3 (1.5)	3.1 (3.1)
# of Years Smoked	16.9 (15.7)	16.2 (18.4)	16.7 (16.6)
Biomass Fuel Use			
Less than or equal to 50% of cooking needs	114 (81.4%)	186 (73.8%)	300 (76.5%)
Greater than 50% of cooking needs	26 (18.6%)	66 (26.2%)	92 (23.5%)
Biomass smoke exposure: hours/day			
Less than or equal to 2 h	79 (56.4%)	149 (59.1%)	228 (58.2%)
Greater than 2 h	61 (43.6%)	103 (40.9%)	164 (41.8%)
Firewood as primary biomass source	129 (92.1%)	213 (84.5%)	342 (87.2%)
Hole in wall/roof in cooking area	125 (89.3%)	225 (89.3%)	350 (89.3%)
Have electricity	50 (35.7%)	99 (39.4%)	149 (38.1%)
Self-report of positive HIV test	2 (1.4%)	5 (2.0%)	7 (2.2%)
Self-report of prior tuberculosis	2 (1.4%)	3 (1.2%)	5 (1.3%)

Data are mean (standard error) or number (%).

*20 sticks = 1 pack of cigarettes.

Asthma was identified in 85 (21.7%) participants, using the question about wheezing or whistling in the last 12 months (Table 2). Wheezing with exercise was reported among 72 (18.7%) of participants and nighttime cough was reported among 71 (18.1%) participants. However, only 33 (8.5%) participants had been previously diagnosed with asthma by a healthcare provider. Mean SGRQ-C scores adjusted to SGRQ was 9.99 (SE 1.00) for men and 16.58 (SE 1.28) for women. Women had higher total scores, as well as symptom (25.17 vs. 20.34), activity (23.38 vs. 13.44) and impact (11.12 vs. 6.63) component scores compared to men. Results stratified by ability to produce acceptable spirometry are shown in Supplementary Appendix SA5.

**TABLE 2:** Symptoms in the last 12 months, St. George’s Respiratory Questionnaire, and fractional exhaled nitric oxide results (Eldoret, Kenya, 2017).

	Male	Female	Total
	(N = 140)	(N = 252)	(N = 392)
Wheezing or whistling in the last 12 months	26 (18.7%) [10.62%–30.81%]	59 (23.4%) [17.32%–30.85%]	85 (21.7%) [15.89%–28.99%]
Number of attacks			
1–3	18 (12.86%) [8.03%–19.96%]	36 (14.29%) [10.20%–19.64%]	54 (13.78%) [10.36%–18.09%]
4–12	6 (4.29%) [1.25%–13.68%]	9 (3.57%) [1.70%–7.34%]	15 (3.83%) [1.81%–7.92%]
>12	2 (1.43%) [0.34%–5.78%]	14 (5.56%) [3.06%–9.89%]	16 (4.08%) [2.24%–7.33%]
Sleep Disturbance in the last 12 months			
<1 night/week	8 (5.71%) [2.64%–11.91%]	17 (6.75%) [3.41%–12.90%]	25 (6.38%) [3.65%–10.90%]
1+ nights/week	5 (3.57%) [1.56%–7.98%]	17 (6.74%) [4.16%–10.75%]	22 (5.61%) [3.75%–8.31%]
Wheezing with exercise in the last 12 months	16 (11.59%) [6.60%–19.57%]	56 (22.58%) [17.04%–29.29%]	72 (18.65%) [14.19%–24.12%]
Nighttime cough in the last 12 months	21 (15.0%) [10.28%–21.36%]	50 (19.84%) [14.24%–26.95%]	71 (18.11%) [13.89%–23.28%]
Ever been diagnosed with asthma by healthcare provider	9 (6.47%) [3.02%–13.33%]	24 (9.68%) [5.75%–15.83%]	33 (8.53%) [5.28%–13.48%]
SGRQ-C QUESTIONNAIRE			
Total Score	7.65 (1.11)	14.97 (1.42)	12.33 (1.11)
Symptoms Score	19.59 (1.59)	24.47 (1.64)	22.72 (1.38)
Activity Score	7.40 (1.50)	18.81 (1.75)	14.71 (1.38)
Impacts Score	5.05 (1.04)	10.16 (1.33)	8.33 (1.01)
SGRQ-C ADJUSTED TO SGRQ**			
Total Score	9.99 (1.00)	16.58 (1.28)	14.20 (1.00)
Symptoms Score	20.34 (1.57)	25.17 (1.62)	23.44 (1.37)
Activity Score	13.44 (1.30)	23.38 (1.52)	19.81 (1.19)
Impacts Score	6.63 (0.91)	11.12 (1.17)	9.51 (0.89)
FeNO	N = 132	N = 237	N = 369
Mean (SE)	27.7 (3.65)	28.8 (2.25)	32.0 (1.96)

Data are number (%) with [95% Confidence Intervals] or mean (standard error).

**For reference, mean St. George’s Respiratory Questionnaire scores in normal subjects with no history of respiratory disease are Total: 6; Symptoms: 12; Activity: 9; and Impacts: 2 [26]. The threshold for a clinically significant difference between groups of patients is 4 units [33].

**FIGURE 1:**
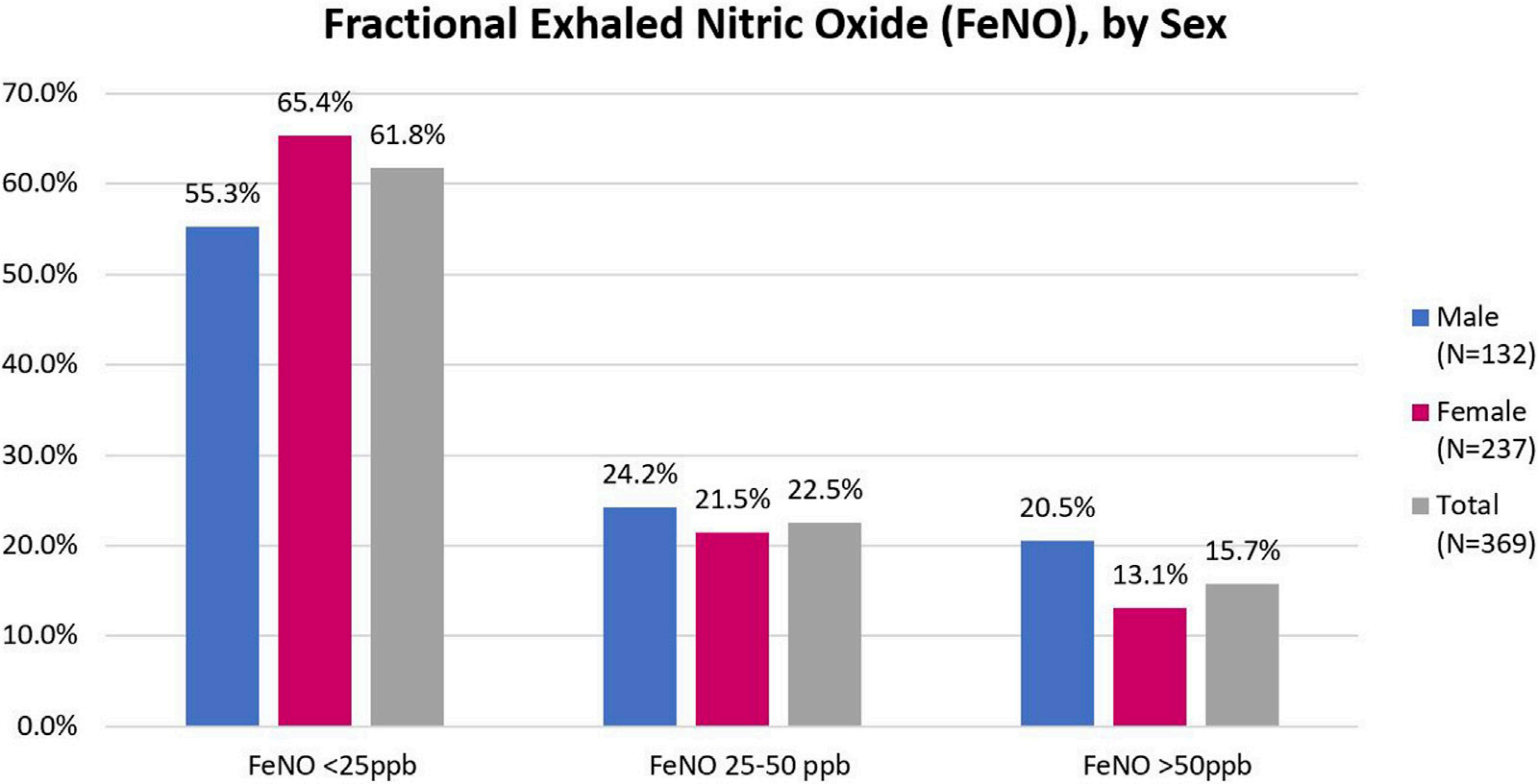
Fractional exhaled nitric oxide (FeNO) by sex (Eldoret, Kenya, 2017).

Spirometry and FeNO results are shown in Table 3 and Figure 1, respectively. The sample prevalence of obstruction on pre-bronchodilator spirometry was 12.3%. Women comprised 62% of the sample population, but over 75% of those with obstruction. Mean age of participants with obstruction was 41.1 years (SD 16.4). Among participants with obstruction, 9 (24.3%) were under the age of 30 years and 12 (35.3%) were 30–39 years. Most participants had mild or moderate airflow obstruction. 24 (71%) patients with obstruction on pre-bronchodilator spirometry completed bronchodilator testing and 8 (33.3%) of those patients met ATS criteria for a positive bronchodilator response. Additionally, 44 (14.4%) participants had no evidence of obstruction, but had an FVC <LLN, suggesting possible underlying restrictive lung disease. Of the 369 individuals who complete FeNO testing, the median FeNO was 22 for men and 17 for women and 58 (15.7%) participants had a FeNO reading of >50 ppb.

**TABLE 3:** Questionnaire and spirometry results (Eldoret, Kenya, 2017).

	Male	Female	Total
	(N = 116)	(N = 189)	(N = 305)
Measured FVC (L), pre-bronchodilator			
Mean (SE)	3.96 (0.81)	3.13 (0.56)	3.45 (0.78)
Min, Max	1.8, 6.3	1.5, 4.8	1.5, 6.3
% Predicted FVC, pre-bronchodilator			
Mean (SE)	88.1 (1.0)	90.5 (0.9)	89.6 (0.7)
Min, Max	60.9, 123.8	59.2, 126.9	59.2, 126.9
FVC Z-score, GLI			
Mean (SE)	−1.03 (0.09)	−0.80 (0.07)	−0.89 (0.06)
Min, Max	−3.4, 1.6	−3.7, 1.8	−3.7, 1.8
Measured FEV1 (L), pre-bronchodilator			
Mean (SE)	3.28 (0.74)	2.50 (0.52)	2.80 (0.72)
Min, Max	1.5, 4.8	1.0, 3.9	1.0, 4.8
% Predicted FEV1, pre-bronchodilator			
Mean (SE)	88.1 (1.2)	85.7 (1.0)	86.6 (0.8)
Min, Max	47.8, 121.3	39.3, 119.8	39.3, 121.3
FEV1 Z-score, GLI			
Mean (SE)	−0.94 (0.09)	−1.12 (0.07)	−1.05 (0.06)
Min, Max	−4.0, 1.4	−3.9, 1.6	−4.0, 1.6
Measured FEV1/FVC Ratio (%), pre-bronchodilator			
Mean (SE)	0.83 (0.08)	0.80 (0.08)	0.81 (0.08)
Min, Max	0.5, 1.0	0.5, 1.0	0.5, 1.0
FEV1/FVC Ratio Z-score			
Mean (SE)	−0.002 (0.11)	−0.73 (0.08)	−0.45 (0.07)
Min, Max	−4.2, 3.2	−4.2, 1.6	−4.2, 3.2
Obstruction on Pre-Bronchodilator Spirometry (FEV1/FVC < LLN)			
Yes, N (%)	8 (6.90%)	29 (15.34%)	37 (12.13%)
Bronchodilator Response*			
Yes, N (%); *n* = 24	2 (33.33%)	6 (33.33%)	8 (33.33%)
Obstruction on Post-Bronchodilator Spiro			
Yes, N (%); *n* = 24	2 (33.33%)	10 (55.56%)	12 (50.00%)
Severity of Obstruction based on Pre-BDR FEV1**			
Mild, N (%)	6 (5.17%)	20 (10.58%)	26 (8.52%)
Moderate, N (%)	1 (0.86%)	8 (4.23%)	9 (2.95%)
Severe, N (%)	1 (0.86%)	1 (0.53%)	2 (0.66%)
FVC <LLN on Pre-Bronchodilator Spiro***			
Yes, N (%)	19 (16.38%)	25 (13.23%)	44 (14.43%)

Data are mean (standard error) or number (%) with [95% Confidence Intervals].

*Bronchodilator testing performed on 24 of 37 patients with obstruction on pre-bronchodilator testing. Bronchodilator response was defined as a >12% improvement in FEV1 or FVC on post-bronchodilator testing as compared to pre-bronchodilator values. 95% confidence intervalss were not reported given small sample size.

**Severity of obstruction was determined using American Thoracic Society/European Respiratory Society Z-score criteria (mild: LLN to −2.50; moderate: −2.51 to −4.0; severe: <4.0.

**Restriction was considered to be suggested if FEV1:FVC ratio was >LLN and FVC was < LLN.

Univariable analysis showed that significant biomass fuel use or high smoke exposure did not differ substantially between participants with airflow obstruction and those without (Table 4). Female sex, age, and BMI were positively associated with higher prevalence of airflow obstruction, but this association was imprecisely estimated. Multivariable regression showed a substantially large crude and adjusted prevalence of obstruction among females and a smaller crude and adjusted prevalence with increasing age, though estimates were relatively imprecise. There was little evidence that prevalence of obstruction was associated with tobacco history. Of note, the intraclass correlation coefficient (ICC) estimate for clustering of obstruction at the household level was 42.1% (95% CI 16.3%–73.0%).

**TABLE 4:** Univariable and multivariable modified poisson regression models for correlates with obstruction on spirometry, *n* = 305 (Eldoret, Kenya, 2017).

Independent variable	Crude model		Multivariable model*	
	Prevalence ratio	(95% CI)	Prevalence ratio	(95% CI)
Sex				
Male	REF			
Female	2.04	0.99, 4.21	1.98	0.94, 4.34
Age, years (Z-score, continuous, linear)	1.22	0.92, 1.63	1.22	0.89, 1.67
Tobacco history (ever)	305			
No	REF			
Yes	0.93	0.37, 2.31	0.93	0.34, 2.52
BMI (Z-score, continuous, linear)	1.14	0.84, 1.53		
Biomass fuel use				
<50% of cooking needs	REF			
>50% of cooking needs	0.89	0.41, 1.93		
Firewood as primary biomass source				
No	REF			
Yes	1.01	0.40, 2.52		
High smoke (hours per day)				
≤2 h	REF			
>2 h	0.72	0.37, 1.39		
Wheezing or whistling in past 12 months, n	304			
No	REF			
Yes	**2.88**	**1.60, 5.19**		
FeNO, categorical, n	291			
<25 ppb	REF			
25–50 ppb	1.52	0.67, 3.45		
>50 ppb	**3.13**	**1.56, 6.27**		

*Confounding adjustment set for Any Obstruction (*n* = 305) includes: age (linear), sex (binary), and tobacco history (binary).

Bolded values are those estimates where the 95% CI does not cross 1.

The presence of wheezing or whistling in the last year was associated with a higher prevalence of airflow obstruction (PR 2.88, 95% CI 1.60–5.19), as was FeNO >50 ppb (PR 3.13, 95% CI 1.56–6.27). Multivariable regression showed that the association between FeNO and obstruction did not change after adjusting for BMI, tobacco use, and firewood use (Supplementary Appendix SA6).

Of the 58 participants with an FeNO >50 ppb, nearly half reported wheezing in the past 12 months (Table 5). The mean SGRQ-C Total score among this group was 18.24. Regression modeling showed a strong association with wheezing or whistling in the last 12 months and FeNO levels >50 ppb (PR 2.93, 95% CI 1.85–4.62) as well as SGRQ-C total scores (PR 1.01, 95% CI 1.00–1.03) and FeNO >50 ppb. These associations persisted after adjusting for age, sex, BMI category, and tobacco use history.

**TABLE 5:** Association between fractional exhaled nitric oxide >50 parts per billion with Wheezing or Whistling in the last 12 months and St. George Respiratory Questionnaire results (Eldoret, Kenya, 2017).

	FeNO >50 ppb (N = 58)	Prevalence ratio univariable model	Prevalence ratio multivariable model
Age	40.5 (2.44)		
Female	31 (53.4%)		
BMI	21.8 (0.78)		
Wheezing or whistling in the last 12 months	26 (44.8%)	2.93 (1.85, 4.62)	3.22 (1.94, 5.32)
SGRQ-C Total Score Unadjusted	16.82 (18.95*)	1.01 (1.00, 1.03)	1.02 (1.00, 1.03)
SGRQ-C Total Score Adjusted	18.24 (2.34*)	1.01 (1.00, 1.03)	1.02 (1.00, 1.03)

Data are number (%) or mean (standard error); Prevalence ratio is shown with (95% confidence interval).

FeNO <50 is reference variable. Confounding adjustment set for FeNO includes: age, sex, BMI (categorical), and history of tobacco smoking (binary).

*Standard error does not account for clustering for SGRQ-C Total Scores because one of the strata (sex) represents a single sampling unit.

## Discussion

This is one of the first cluster-randomly sampled, cross-sectional surveys to estimate the prevalence and burden of asthma and airflow obstruction and its correlates in Kenya. The prevalence of asthma in our sample is estimated to be 21.7%. The sample prevalence of spirometry-defined airway obstruction is estimated to be 12.3% and the sample prevalence of eosinophilic airway inflammation is estimated to be 15.7%. Participant report of wheezing or whistling in the last year was associated with a higher prevalence of airway obstruction on spirometry. This specific question may be useful in identifying patients with airflow obstruction in Kenya. Our sample population had more females (64.3%) compared to the population of females in Uasin Gishu during the 2019 Kenyan Census (50.1%), and had a greater proportion of participants at ages 40 years and above. Given that female sex and older age were both positively associated with obstruction in our sample, our estimates may be higher than the population prevalence. However, our data highlights the burden of airway disease and airflow obstruction among women and in a relatively young population with low rates of tobacco use.

Our sample population was notably younger compared to other population-based studies such as Burden of Lung Disease (BOLD) [39]. However, there was little evidence that the prevalence of obstruction varied by age, sex, BMI, tobacco history, significant biomass fuel use, firewood use, or high smoke exposure. The lack of an association between tobacco use and obstruction may be explained by the relatively low mean pack-year history among participants. We hypothesize that we did not detect a difference in our prevalence estimates in relation to biomass exposure in part due to lack of precise measures such as particulate matter concentrations. Notably, the ICC estimate at the household level was 32.1%, suggesting that 32.1% of the variation in obstruction occurs within the household. This suggests that people in the same household have a similar risk of obstruction; that risk may be due to environmental factors such as biomass fuel use. This interpretation is limited by the wide confidence interval of 7.8%–72.5% and that the ICC may incorporate clustering at the village level.

Mean SGRQ scores for both men and women were notably higher than established mean scores in normal subjects with no history of respiratory disease. They were also higher among women as compared to men, which is in contrast to our findings that there were no major differences in prevalence estimates of airway obstruction or in FeNO values between sexes. While the utility of SGRQ in individuals without respiratory disease may be limited, studies establishing population norms for SGRQ scores and validating the SGRQ and SGRQ-C did not demonstrate this degree of sex difference nor did they find such high values for those without known airway disease [40–43]. However, studies specifically analyzing sex and quality of life among patients with COPD found worse quality of life among women, highlighting the importance of further exploring sex-specific determinants of quality of life, especially among those with lung disease in East Africa [44, 45].

Prior studies have shown that FeNO levels, either alone or in combination with blood eosinophil counts, have high diagnostic specificity for eosinophilic predominant airway inflammation, and that a cutoff of >50 ppb can indicate a high likelihood of corticosteroid responsiveness in symptomatic individuals [46, 47]. We found that participants with FeNO levels of >50 ppb were 3.6-fold more likely to indicate wheezing within the last 12 months and 3-fold more likely to have obstruction on spirometry, suggesting FeNO testing may be a useful diagnostic tool in settings where spirometry is limited, providing a point of care method to identify patients who may benefit from corticosteroids [48, 49]. Importantly, FeNO can be reduced by tobacco use or smoke exposure and the effects of biomass exposure on FeNO is not clear [50, 51]. Multivariable analysis did not show a change in the association between FeNO>50 ppb and obstruction when adjusting for tobacco use, BMI or firewood use.

Finally, we note a higher sample prevalence of subjects with pre-bronchodilator FVC <LLN as compared to the FRESH AIR and BOLD studies, underscoring the need for further lung volume assessment capabilities in Kenya [7, 52]. Reduced FVC while suggestive of restriction, may also be due to poor effort. As formal lung volume measurements were unable to be obtained, true restrictive impairment could not be fully assessed.

Strengths of our study include rigorous sampling methodology which combined GIS mapping with a standardized three-stage sampling, allowing for probability sampling of individuals across the rural to urban spectrum. Limitations include that we did not reach our target sample size which likely reduced the precision of our estimates. Sampling was conducted at individual homes during normal work hours. The probability of inclusion thus may be biased based on who is more likely to be home at those times and may explain why our sampled population included a higher proportion of women. There is also the potential for measurement error especially for self-reported variables. Given HIV and tuberculosis related stigma, we suspect our data likely are an under-representation of the true prevalence of these diseases in this population (HIV rates are between 4.3%–5.6% in Uasin Guishu county) [53]. Furthermore, we did not collect direct measurements of indoor air pollution which could have better quantified individual exposure. After recalculating predicted values for spirometry according to GLI equations, there were a relatively low proportion of participants who underwent bronchodilator testing. Measurement error in spirometry may threaten the validity of our prevalence estimates, and measurement error of other variables, if differential by outcome status, may threaten the validity of our estimated prevalence ratios. However, there is little reason to believe that a variable in our study was differentially measured by level of obstructive airway disease. Finally, given the cross-sectional nature of our study, we cannot rule out reverse causation; however, for our associations, this seems unlikely.

Obstructive lung disease is a significant health problem in Kenya affecting a young population who lack a significant tobacco use history. Further research is needed to understand the prevalence of airflow obstruction in Kenya, its associated risk factors, and most appropriate management. In areas where spirometry is unavailable, further evaluation of using validated questionnaires and FeNO testing for diagnosis and treatment should be conducted. Ultimately, greater infrastructure for the assessment, diagnosis and treatment of lung disease in Kenya is needed.
