# Supplementary figures and images for: Asthma, Airflow Obstruction, and Eosinophilic Airway Inflammation Prevalence in Western Kenya: A Population-Based Cross-Sectional Study
Source: Int J Public Health. 2023 Aug 17;68:1606030. doi: 10.3389/ijph.2023.1606030 (PMC10468572; doi:10.3389/ijph.2023.1606030)

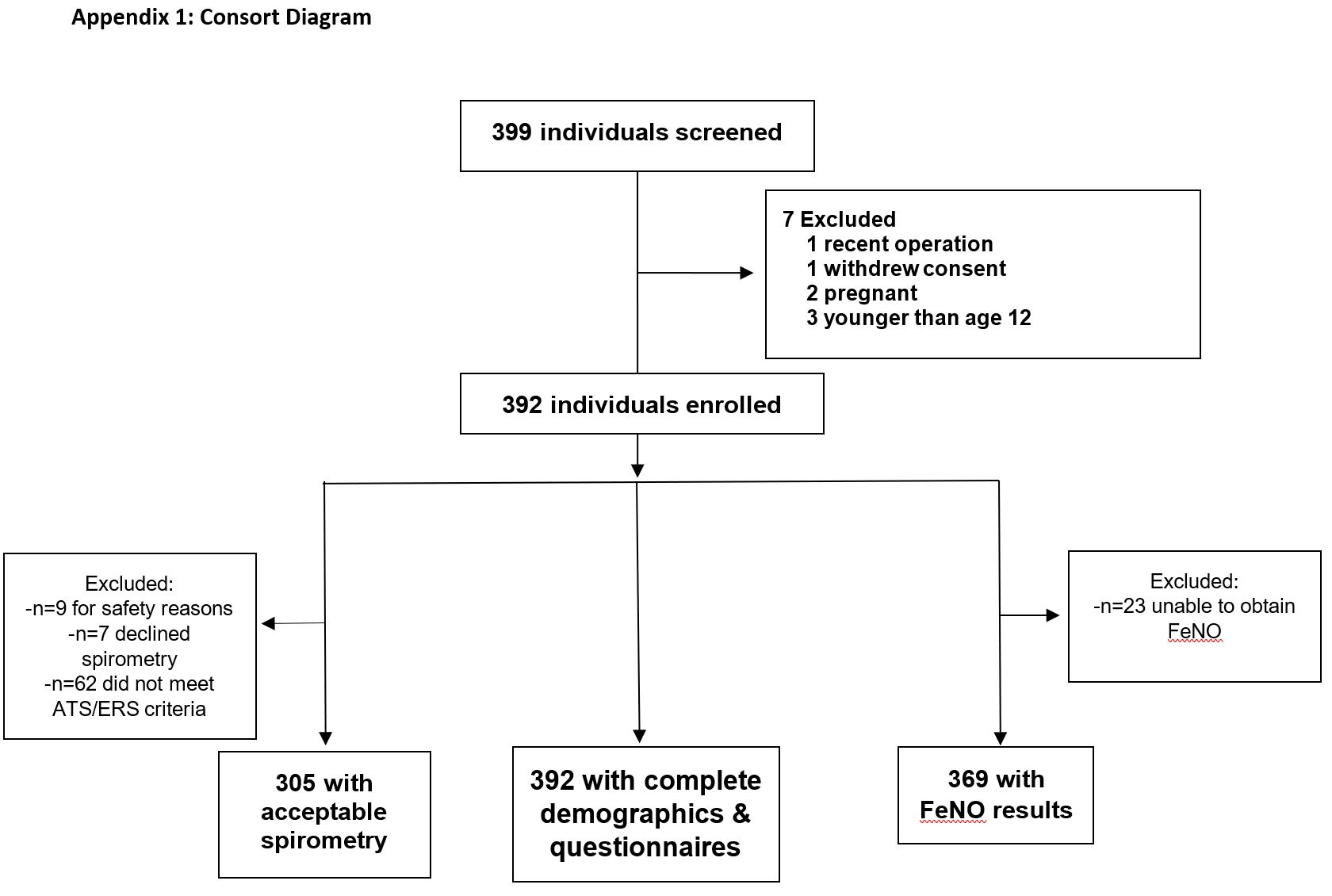

Supplement: Supplementary file 1 [file Image1.JPEG]
