# Supplementary material for: Asthma, Airflow Obstruction, and Eosinophilic Airway Inflammation Prevalence in Western Kenya: A Population-Based Cross-Sectional Study
Source: Int J Public Health. 2023 Aug 17;68:1606030. doi: 10.3389/ijph.2023.1606030 (PMC10468572; doi:10.3389/ijph.2023.1606030)
Supplement: Supplementary file 2 [file DataSheet1.docx]

**Supplementary Materials:**

**Appendix 2: List of Administrative Sub-Units around Eldoret Selected for Sampling**

| **Location:** | **Setting:** | **Longitude:** | **Latitude:** |
| --- | --- | --- | --- |
| CHEPKOILEL | urban/periurban/rural | 0.559514 | 35.287366 |
| CHEPTIRET | rural | 0.380594 | 35.341702 |
| KAPSARET | rural | 0.45633 | 35.15344 |
| KAPSINENDE | rural | 0.452467 | 35.388628 |
| KAPSOYA | urban/periurban/rural | 0.542644 | 35.339389 |
| KAPTAGAT | rural | 0.482996 | 35.376944 |
| KAPYEMIT | urban/periurban | 0.569387 | 35.236834 |
| KIBULGENY | urban/periurban | 0.560743 | 35.282039 |
| KIPCHAMO | rural/periurban | 0.493352 | 35.306771 |
| KIPLOMBE | rural | 0.653676 | 35.214649 |
| MEGUN | rural | 0.334645 | 35.236938 |
| MUMETET | rural | 0.726813 | 35.345059 |
| NGERIA | rural/periurban | 0.379055 | 35.274685 |
| PIONEER | urban/periurban | 0.474594 | 35.25993 |
| SERGOIT | rural | 0.581016 | 35.336525 |
| SIMAT | rural/periurban | 0.546941 | 35.202408 |
| SUGOI | rural | 0.555172 | 35.110036 |
| TAPSAGOI | urban/periurban | 0.572412 | 35.016955 |
| TEMBELIO | rural | 0.52519 | 35.362541 |

**Appendix 3: Population of Uasin Gishu and Study Sample by Age**

| Age | Uasin Gishu Population* | Sample Population |
| --- | --- | --- |
| 12-14 | 2.5% | 5.9% |
| 15-19 | 11.3% | 11.00% |
| 20-24 | 11.0% | 14.0% |
| 25-29 | 9.1% | 12.0% |
| 30-34 | 8.0% | 12.5% |
| 35-39 | 5.9% | 7.4% |
| 40-44 | 4.9% | 8.2% |
| 45-49 | 3.9% | 4.8% |
| 50-54 | 2.6% | 4.1% |
| 55-59 | 2.1% | 4.3% |
| 60-64 | 1.5% | 4.3% |
| 65-69 | 1.1% | 3.6% |
| 70-74 | 0.7% | 2.3% |
| 75-79 | 0.5% | 1.8% |
| 80-84 | 0.3% | 0.8% |
| 85-89 | 0.2% | 1.3% |
| 90-94 | 0.1% | 1.8% |

*****Source: Kenyan National Bureau of Statistics, 2019 Census

**Appendix 4: Clinical Characteristics**

| \|  \| Male \| Female \| Total \| \| --- \| --- \| --- \| --- \| \|  \| (N = 140) \| (N = 252) \| (N = 392) \| |
| --- | --- | --- | --- | --- | --- | --- | --- | --- |
| \| **Measured Heart Rate (bpm)** \| 71.2 (1.2) \| 77.9 (0.7) \| 75.5 (0.7) \| \| --- \| --- \| --- \| --- \| \| **Measured Systolic Blood Pressure** \| 127 (1.4) \| 123 (1.1) \| 125 (1.0) \| \| **Measured Diastolic Blood Pressure** \| 80 (1.2) \| 78 (0.7) \| 79 (0.7) \| \| **Measured Pulse Oximetry (SaO2%)** \| 96 (0.3) \| 95 (0.2) \| 95 (0.2) \| \| **Positive self-report of prior HIV testing** \| 108 (77.1%) \| 216 (85.7%) \| 324 (82.7%) \| \| **Self-report of prior heart disease** \| 1 (0.7%) \| 5 (2.0%) \| 6 (1.5%) \| \| **Self-report of prior hospitalizations** \| 41 (29.3%) \| 83 (32.9%) \| 124 (31.6%) \| \|  \|  \|  \|  \|   *Data are mean (SE) or number (%)* |

**Appendix 5: Symptoms in the Last 12 Months, SGRQ-C, and FeNO Results, stratified by Ability to Produce acceptable spirometry**

| \|  \| **Met ATS Criteria** \| **Did Not Meet ATS Criteria** \| \| --- \| --- \| --- \| |
| --- | --- | --- | --- |
| \| **SYMPTOMS** \| N = 305 \| N = 62 \| \| --- \| --- \| --- \| \| **Wheezing or whistling in the last 12 months** \| 61 (20.0%) \| 17 (27.4%) \| \|  \|  \|  \| \| **Number of attacks** \|  \|  \| \| 1-3 \| 37 (12.1%) \| 13 (21.0%) \| \| 4-12 \| 11 (3.6%) \| 3 (4.8%) \| \| >12 \| 13 (4.3%) \| 1 (1.6%) \| \|  \|  \|  \| \| **Sleep Disturbance in the last 12 months** \|  \|  \| \| <1 night/week \| 17 (5.6%) \| 5 (8.1%) \| \| 1+ nights/week \| 17 (5.6%) \| 4 (6.5%) \| \| **Wheezing with exercise in the last 12 months** \| 55 (18.0%) \| 13 (21.0%) \| \|  \|  \|  \| \| **Nighttime cough in the last 12 months** \| 53 (17.4%) \| 12 (19.4%) \| \|  \|  \|  \| \| **Ever been diagnosed with asthma by health care provider** \| 26 (8.5%) \| 5 (8.1%) \| \|  \|  \|  \| \| **SGRQ-C QUESTIONNAIRE** \| N = 303 \| N=62 \| \| Total Score \| 13.44 (0.77) \| 15.48 (2.28) \| \| Symptoms Score \| 21.97 (1.02) \| 25.24 (2.65) \| \| Activity Score \| 13.51 (1.20) \| 17.19 (3.20) \| \| Impacts Score \| 7.48 (0.75) \| 10.07 (2.26) \| \|  \|  \|  \| \| **FeNO** \| N=291 \| N=56 \| \| Mean (SE) \| 31.61 (2.03) \| 35.04 (7.31) \| \|  \|  \|  \|   *Data are number (%) or mean (SE)* |

**Appendix 6: Multivariable Modified Poisson Regression Models for Correlates with FeNO and Obstruction on Spirometry, n=291.**

|  |  | **Crude Model** | |  | **Multivariable model*** | |
| --- | --- | --- | --- | --- | --- | --- |
| **Independent variable** | | **Prevalence Ratio** | **(95% CI)** |  | **Prevalence Ratio** | **(95% CI)** |
| FeNO, categorical | |  |  |  |  |  |
| <25 ppb | | REF |  |  |  |  |
| 25-50 ppb | | 1.42 | 0.59, 3.42 |  | 1.10 | 0.42, 2.92 |
| >50 ppb | | 3.36 | 1.65, 6.84 |  | 3.36 | 1.66, 6.82 |
| Tobacco history (ever) | | 305 |  |  |  |  |
| No | | REF |  |  |  |  |
| Yes | | 1.03 | 0.41, 2.59 |  | 0.52 | 0.14, 1.90 |
| BMI (Z-score, continuous linear) | | 1.13 | 0.82, 1.55 |  | 1.14 | 0.79, 1.64 |
| Firewood as primary biomass source | |  |  |  |  |  |
| No | | REF |  |  |  |  |
| Yes | | 1.21 | 0.43, 3.44 |  | 1.04 | 0.37, 2.95 |
|  | |  |  |  |  |  |
|  | |  |  |  |  |  |
